# A comparative method for protein extraction and 2-D gel electrophoresis from different tissues of *Cajanus cajan*

**DOI:** 10.3389/fpls.2015.00606

**Published:** 2015-08-07

**Authors:** Nisha Singh, Neha Jain, Ram Kumar, Ajay Jain, Nagendra K. Singh, Vandna Rai

**Affiliations:** Functional Genomics, National Research Centre on Plant Biotechnology, New Delhi, India

**Keywords:** pigeonpea, proteomics, SDS PAGE, 2-DE, tissue-specific

## Abstract

Pigeonpea is an important legume crop with high protein content. However, it is often subjected to various abiotic and biotic stresses. Proteomics is a state-of-the-art technique used to analyze the protein profiling of a tissue for deciphering the molecular entities that could be manipulated for developing crops resistant to these stresses. In this context, developing a comprehensive proteome profile from different vegetative and reproductive tissues has become mandatory. Although several protein extraction protocols from different tissues of diverse plant species have been reported, there is no report for pigeonpea. Here, we report tissue-specific protein extraction protocols representing vegetative (young leaves), and reproductive (flowers and seeds) organs and their subsequent analysis on 2-dimensional gel electrophoresis. The study explicitly demonstrated that the efficacy of a particular protein extraction protocol is dependent on the different tissues, such as leaves, flowers and seeds that differ in their structure and metabolic constituents. For instance, phenol-based protocol showed an efficacy toward higher protein yield, better spot resolution and a minimal streaking on 2-DE gel for both leaves and flowers. Protein extraction from seeds was best achieved by employing phosphate-TCA-acetone protocol.

## Introduction

India is the largest consumer and producer of Pigeonpea (*Cajanus cajan*), a tropical legume, for its dietary properties. It is also being cultivated in other tropical and subtropical countries (Myanmar, Kenya, Malwai, Tanzania, Uganda, and Nepal) and enriches soil through symbiotic nitrogen fixation. In India, it is largely known as arhar or tur with an area of 4.6 mha and productivity is 762.4 kg ha^–1^ respectively (FAOSTAT, 2015). Due to high protein content (18–30%) and its ease of digestibility (68%), it is a major source of proteins particularly for a large section of Indian population that strictly depends on vegetarian meals (Reddy et al., 1979; Chitra et al., 1996; Sharma et al., 2011). In this context pigeonpea has become a priority crop in India with an effort toward developing high yielding varieties by conventional breeding approach and/or biotechnological interventions. These approaches are important more so because it is a hardy crop and is often subjected to various biotic and abiotic stress (Varshney et al., 2010). The generation of proteomic atlas employing state of the art technologies such as 2-DE, MALDI, iTRAQ, isotope coded affinity tag (ICAT) and orbitrap are increasingly becoming attractive paradigm for identification of novel proteins (Agrawal et al., 2013; Zargar et al., 2015). This has expedited the potential exploitation of these candidate proteins by complementary omic approaches. Pigeonpea is the first seed legume to have its complete genome sequenced through global research consortium (Singh et al., 2012; Varshney et al., 2012). This has provided further fillip toward developing this legume that would be tolerant to various biotic and/or abiotic stresses and exhibiting high yield potential under adverse agro-climatic conditions.

Different vegetative and reproductive tissues are endowed with different metabolomes that are developmentally regulated in a species-specific manner. Further, cellular proteins from different tissues often vary in their properties with respect to their charge, size, proteolysis, hydrophobicity, ligand interactions and localization (Isaacson et al., 2006). In addition, plant tissues generally contain low amount of proteins with high proteases, which limits tissue dissolution and subsequent isoelectric focusing (IEF; Wu and Wang, 1984). Likelihood of developing a common protocol for protein extraction from different tissues of taxonomically diverse plants species has often met with only a limited success. Therefore, there have been global efforts for developing easy-to-use protocol for optimal extraction of proteins from different tissues of diverse plant species (Saravanan and Rose, 2004; Isaacson et al., 2006; Wang et al., 2008). Overall, the general consensus is that TCA-acetone based protein extraction is effective for younger tissues (Damerval et al., 1986; Santoni et al., 1997). While phenol extraction followed by ammonium acetate precipitation in methanol is considered more suitable for rather recalcitrant tissues (Wang et al., 2003; Carpentier et al., 2005). However, it remains a matter of conjecture whether protocols developed for young and/or recalcitrant tissues for different species could be successfully employed for generating a comprehensive proteomic atlas of pigeonpea from its vegetative and reproductive tissues.

Therefore to determine whether tissue-specific metabolic constituents of pigeonpea necessitate specific protein extraction protocol, in the present study different methods of protein extraction (phenol, TCA-acetone and phosphate-TCA-acetone) were evaluated for mature leaf, flowers and seeds collected from field-grown pigeonpea. The results explicitly demonstrated the efficacy of different protein extraction protocols dependent on the metabolic constituents of the tissues. Further, merits of these tissue-specific protein extraction methods on quantitative and qualitative properties of the extracted proteins and their discrete resolution during IEF are discussed.

## Materials and Methods

### Plant Material

Pigeonpea (*Cajanus cajan*, variety ASHA, ICPL-87119) was used in the present study. Various tissue samples (green leaf and fully opened flower) were collected from the plants grown and maintained by Department of Genetics, Indian Agricultural Research Institute, New Delhi after 8 months of sowing. Seeds were taken from fully mature plant, i.e., after 9 months of sowing. Harvested tissues were preserved at –80°C till further use.

### Reagents

The chemicals and the reagents used in the experiments were HPLC grade and procured from Sigma-Aldrich (≥99.93%). Water from a Millipore Milli-RO4 reverse osmosis system was used for preparing all the solutions.

2-Mercaptoethanol (2-ME, Sigma-Aldrich, cat. no. M6250), 3-[(3-Cholamidopropyl) dimethylammonio]-1-propanesulfonate (CHAPS, Sigma-Aldrich, cat. no. C3023), Acetone (Sigma-Aldrich, cat. no. 650501), Acetic acid (Sigma-Aldrich, cat. no. V800018), Agarose (Sigma-Aldrich, cat. no. A9539), Ammonium acetate (Sigma-Aldrich, cat. no. A7262), Bradford reagent (Sigma-Aldrich, cat. no. B6916), Bromophenol blue (BPB, Sigma-Aldrich, cat. no. B0126), Bovine serum albumin (BSA, Sigma-Aldrich, cat. no. 05470), Coomassie brilliant blue (CBB, Bioworld, cat. no. 742083), Dithiothreitol (DTT, Sigma-Aldrich, cat. no. D9779), Ethylene diamine tetra-acetic acid, 0.5 M, pH 8.0 (EDTA, Sigma-Aldrich, cat. no. E7889), Glycerol (Sigma-Aldrich, cat. no. G5516), Glycine (Sigma-Aldrich, cat. no. 50046), Hydrogen chloride (HCl, Sigma-Aldrich, cat. no. H1758), Immobiline^TM^ Dry strip, pH 3-10, 13 cm (GE Healthcare, UK, cat. no. 17-6001-14), IPG buffer, pH 3-10 NL (GE Healthcare, UK, cat. no. 17-6000-88), Iodoacetamide (IAA, Sigma-Aldrich, cat. no. I3750), Methanol (Sigma-Aldrich, cat. no. 322415), Phenyl methane sulfonyl fluoride (PMSF, Sigma-Aldrich, cat. no. P7626), Potassium chloride (KCl, Sigma-Aldrich, cat. no. P9541), Phosphate buffer, 1M, pH 7.5 (Sigma-Aldrich, cat. no P3619), Complete EDTA free Protease inhibitor cocktail (Roche, cat. no. REF 11 875 580 001), Sodium dodecyl sulfate (SDS, Sigma-Aldrich, cat. no. L4390), Sucrose (Sigma-Aldrich, cat. no. S9378), Thiourea (Sigma-Aldrich, cat. no. T8656), Trichloroacetic acid (TCA; Sigma-Aldrich, cat. no. 522082), Tris (Trizma base, Sigma-Aldrich, cat. no. T1503), Tris-buffered phenol solution (stored at 4°C, pH 7.8–8.0; Sigma-Aldrich, cat. no. P 4557), Tris-HCl, 1.5 M, pH 8.8 (Amresco, cat. no. M195), Urea (Sigma-Aldrich, cat. no. U6504).

### Extraction Buffers

Protein extractions from different tissues were carried out using three extraction/ buffers:

• Phenol buffer
  Sucrose (0.7 M), Tris (0.5 M), HCI (30 mM), EDTA (50 mM), KCl (0.1 M).
• TCA-Acetone buffer
  TCA (10%, w/v) in acetone with 2-mercaptoethanol (0.07%).
• Phosphate-TCA-Acetone buffer
  Phosphate buffer (0.1 M, pH 7.5), TCA (10%, w/v) in acetone with 2-mercaptoethanol (0.07%).

### Protein Extraction

#### Protocol 1 (Phenol Extraction)

Extraction protocol for membrane proteins from the barley roots (Hurkman and Tanaka, 1986) was modified for extraction of proteins from 1 gm each of fresh tissues (green leaves and fully opened flowers) and mature seeds of pigeonpea. Tissues were pulverized in pre-chilled pestle and mortar using liquid nitrogen and the powder was resuspended in 3 ml of extraction buffer and stored at 4°C. Just prior to use, 2-ME and 1 mM PMSF were added to pre-chilled buffer. The suspension was incubated for 10 min with continuous shaking at 4°C. An equal volume of Tris-buffered phenol was then added and the solution was again incubated on a shaker for 10 min at 4°C. The aqueous and organic phases were separated by centrifugation at 13,000 rpm for 15 min at 4°C. The phenolic phase was carefully recovered and re-extracted with equal volume of extraction buffer. The samples were vigorously vortexed and centrifuged for phase separation at 13,000 rpm for 15 min at 4°C. The phenolic layer was transferred to a fresh tube for precipitation of proteins by addition of ammonium acetate (0.1 M) in cold methanol with subsequent overnight incubation at –20°C. The precipitate/protein pellet was washed thrice with precipitation solution (stored at –20°C) and final washing was done with pre-chilled pure acetone. The pellet was air-dried and then dissolved in rehydration buffer (7 M urea, 2 M thiourea and 2% (w/v) CHAPS).

#### Protocol 2 (Trichloroacetic Acid-Acetone Buffer)

Samples were grounded similarly as described for Protocol 1 and then homogenized with 3 ml solution comprising TCA (10%) in acetone with 2 ME (0.07%). The total protein was precipitated overnight at –20°C. The precipitate was vortexed and centrifuged at 13,000 rpm at 4°C for 15 min. The pellet obtained was rinsed thrice with acetone supplemented with 2 ME (0.07%), EDTA (2 mM) and 1 tablet of complete EDTA free protease inhibitor. For every washing, 500 μl of chilled wash buffer was added, vortexed briskly and centrifuged at 13,000 rpm at 4°C for 15 min. Final washing was carried out with pre-chilled acetone (100%). Air-dried pellet was kept overnight at –80°C for removing remaining traces of acetone. The pellet was then dissolved in rehydration buffer.

#### Protocol 3 (Phosphate-TCA-Acetone Buffer)

The tissue samples were finely grounded using liquid nitrogen. The powdered samples were then homogenized using phosphate buffer (0.1 M, pH 7.5). The supernatant was transferred to a fresh tube containing 2 ml of TCA (10%) in acetone with 2 ME (0.07%) for protein precipitation. The protein pellet was obtained after centrifugation at 13,000 rpm at 4°C for 15 min. The pellet was purified by washing thrice with chilled acetone supplemented with 2 ME (0.07%), EDTA (2 mM) and 1 tablet of complete EDTA free protease inhibitor. Final washing was done using pure acetone. Pellet was kept at –80°C for overnight. Acetone-free pellet was dissolved in rehydration buffer and quantified.

### Protein Quantification

The dried pellet was re-suspended in rehydration buffer and sonicated for 10 min. Insoluble particles were removed by centrifugation at 14,000 rpm for 10 min at 4°C. Proteins isolated from different tissues using Protocols 1–3 were quantified as described (Bradford, 1976).

### Two Dimensional Polyacrylamide Gel Electrophoresis

Protein samples (250 μg of protein in 250 μl of rehydration buffer) containing IPG buffer (0.5%, pH 3–10) were loaded in re-swelling tray. Immobilized linear pH gradient strips (pH 3–10, 13 cm, GE Healthcare, UK) were rehydrated overnight. IEF was carried out using a Ettan IPGPhor3 Isoelectric Focusing System (GE Healthcare, UK) under the following conditions: 100 V for 1 h in step and hold, 500 V for 2 h in step and hold, 1000 V for 1 h in gradient mode, 8000 V for 2.30 h in gradient mode and 8000 V for 30 min in step and hold. After IEF, the strips were incubated for 15 min each in SDS equilibration buffer (6 M urea, 75 mM Tris HCl, pH 8.8, glycerol 29.3%, SDS 2%, BPB 0.002%) first supplemented with DTT (10 mg ml^–1^) and then replaced with equilibration buffer containing IAA (25 mg ml^–1^). The equilibrated strips were then placed on a 12.5% Tris-glycine SDS-PAGE (18 × 16 cm) as described (Laemmli, 1970). The strips were sealed with sealing solution [laemmli buffer, agarose (0.5%) and bromophenol blue (0.002%)]. Second dimension electrophoresis was carried out using Hoefer SE 600 Ruby electrophoresis unit (GE Healthcare, UK) at 30 mA/gel at 25°C. The gels were stained overnight with CBB solution as described (Newsholme et al., 2000) and then destained with solution containing acetic acid 10% and methanol 35% for 8 h.

### Gel Image Analysis

All 2-D CBB stained gels were scanned with high resolution scanner (GE Image Scanner III). Gel images (mel format) were analyzed using ImageMaster 2-D Platinum V.7.0 software (GE Healthcare, UK). For optimal clarity, protein spots were detected by adjusting different parameters, i.e., smoothness, saliency and minimum area. Spots detected near the edges of the gel were deleted manually to avoid erroneous interpretation. 2-D gel match sets were grouped into classes according to the task of analysis. Subsequently, each of the identified spots were marked and numbered. The matches with ANOVA value <0.05 were considered to be significant for analysis and the total number of protein spots in each sample were documented. The data for the proteins extracted by Protocols 1–3 from different tissues were generated from technical triplicates.

## Results and Discussion

**FIGURE 1:**
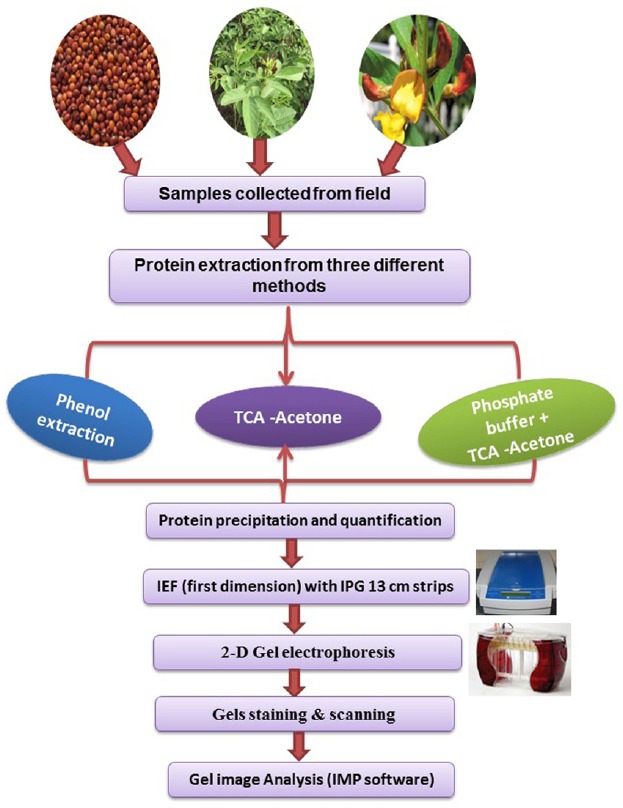
**Flow chart depicting the steps of protein extraction from seed, leaf and flower using three different protocols**.

In earlier studies, different protein extraction protocols have been employed for various tissues from different species. For instance, phenol extraction for plasma membrane protein from barley (Hurkman and Tanaka, 1986), TCA-acetone for leaves of rice, *Arabidopsis*, maize and cucumber (Cho et al., 2006). It was apparent from these studies that a protocol for protein extraction need to be tissue and/or species-specific. However, no protocol has yet been reported for optimal extraction of high quality proteins from different tissues of vegetative and reproductive organs of pigeonpea. Therefore, in this study we attempted to define a protocol suited best for each pigeonpea tissue by comparing the yield and resolution of the protein spots on 2-D gel obtained by following different extraction protocols. Protein extraction using three different methods, from vegetative (leaves) and reproductive (flowers and seeds) tissues collected from field-grown pigeonpea were optimized. Since the metabolic constituent were expected to be tissue-specific, the probability of a common protein extraction protocol providing optimal high quality protein yield from all these ontogenetically diverse tissues was assumed to be improbable. Therefore, in this study, desired tissues were weighed and homogenized and further subjected to different procedures of extraction based on the buffers used (Protocol 1, 2, and 3). The flow diagram (Figure 1) presented here showed different steps involved for proteomics comprising protein precipitation, quantification and iso-electric focusing, 2-D gel electrophoresis, gel staining and scanning and finally image analysis. Proteins are conventionally extracted by aqueous buffer, detergents, or direct precipitation (Michaud and Asselin, 1995). For proteomic studies, other than commonly used trichloro acetic acid (TCA)/acetone precipitation method (Cho et al., 2006), phenol extraction followed by methanol/ammonium acetate precipitation has also been shown to efficient (Hurkman and Tanaka, 1986).

**FIGURE 2:**
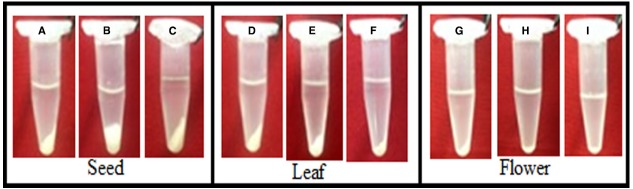
**Tissue-specific protein pellets using Phenol, P+TCA acetone and TCA acetone protein extraction methods.** The tissues of mature seed, green leaf and fully opened flower of pigeonpea variety ASHA were ground to a fine powder in liquid nitrogen and then rinsed with Phenol **(A,D,G)**, P+ TCA acetone **(B,E,H)** and TCA acetone **(C,F,I)**.

In the present study quality and quantity of proteins extracted using Protocols 1–3 from different tissues were determined and compared. Irrespective of the tissue used for protein extraction, protocol based on phenol extraction gave optimal yield compared with other two protocols in vegetative and reproductive tissues (Table 1, Figure 2). The phenol extraction method has normally being used for extracting proteins from recalcitrant tissues from diverse plant species (Wang et al., 2003; Saravanan and Rose, 2004; Carpentier et al., 2005). Earlier studies reported the efficacies of TCA/acetone and phenol-based protocols for extracting proteins from recalcitrant tissues with the latter method was relatively more efficient in removing interfering substances (Saravanan and Rose, 2004; Carpentier et al., 2005).

**TABLE 1:** **Protein yields and spot numbers from pigeonpea seeds, leaves and flowers with phenol, P+TCA-acetone and TCA-acetone extraction methods**.

	**Seed**		**Leaves**		**Flowers**	
**Methods**	**Protein yield (mg/g)**	**Number of spots**	**Protein yield (mg/g)**	**Number of spots**	**Protein yield (mg/g)**	**Number of spots**
Phenol	2.81 ± 0.31	418 ± 12	2.12 ± 0.20	408 ± 15	1.05 ± 0.052	240 ± 15
P+ TCA-acetone	2.12 ± 0.22	480 ± 18	1.54 ± 0.31	380 ± 10	0.035 ± 0.02	154 ± 10
TCA-acetone	1.8 ± 0.1	286 ± 12	1.03 ± 0.24	268 ± 17	0.028 ± 0.21	105 ± 8

Values are the mean of three independent replicates ± standard deviation.

Proteins extracted using different methods differ in their solubility. For instance phenol method facilitate extracting soluble proteins, whereas TCA method is relatively more suitable for extracting total protein including insoluble ones (Carpentier et al., 2005, e.g., seed storage proteins of the globulin family). In this context, addition of phosphate buffer to TCA-Acetone helped in maintaining pH of the solution, which resulted in extraction of high quality protein from seeds.

**FIGURE 3:**
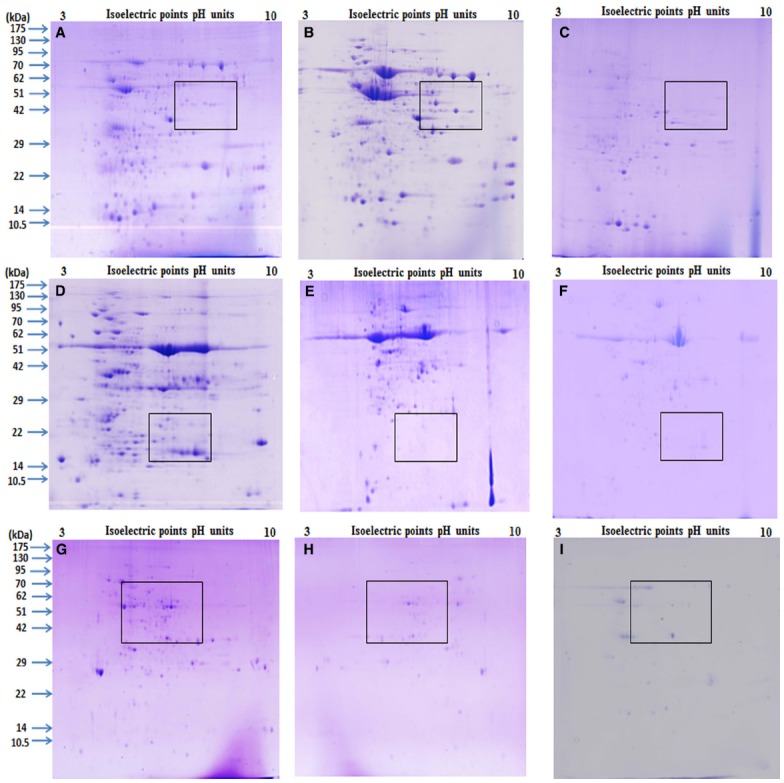
**Comparison of 2 DE gels produced using Phenol, P+TCA acetone and TCA acetone extraction method.** The tissues of mature seed, green leaf and fully opened flower of pigeonpea variety ASHA were ground to a fine powder in liquid nitrogen and then rinsed with Phenol **(A, D,G)**, P+ TCA acetone **(B,E,H)** and TCA acetone **(C,F,I)**. The final proteins were dissolved in the rehydration buffer. Equal protein was loading (approximately 250 μg) resolved by IEF using 13 cm linear IPG strips (pH 3–10) and then separated using 12.5% SDS gels. Gels were stained with CBB. Visible differences marked by rectangles comparison based on: Phenol, P+TCA acetone and TCA acetone protein extraction methods.

After extracting proteins from different tissues using Protocols 1, 2, and 3, they were then separated by 2-DE under identical conditions in the linear 3 to 10 pH range. A comparison was made based on spot focusing and resolution, number of resolved spots and intensity of spots. Although proteins extracted using Protocols 1 and 3 gave better results compared with Protocol 2 (Figure 3), and Protocol 1 and 3 were found to be better suited for relatively soft (leaves and flowers) and hard (seeds) tissues, respectively. However, Protocol 2 yielded a light yellow colored pellet with flowers, hence not suitable for 2-DE (Wu et al., 2014). Notably, Protocol 1 could not only purify the proteins from leaves and flowers that are enriched with phenolic compounds and flavonoids (Stalikas, 2007) but also had an ability for a better cleanup, which lead to higher yield of glycoprotein (Saravanan and Rose, 2004). Lower number of spots observed with Protocol 2 could be due to the presence of impurities in these protein samples that could have possibly contributed toward some horizontal and vertical streaking in these gels. Further the total number of protein spots detected on the gel were compared for seeds, leaves and flowers that were processed by Protocols 1, 2, and 3 (Table 1). Protocol 1 yielded higher number of spots both for leaves and flower compared with Protocols 2 and 3. A clear difference was reported in the pattern of protein spots using different methods in seeds, leaves and flowers as depicted in boxed area of Figure 3. Our results were consistent with an earlier study, which also reported higher efficacy of Protocol 1 in extracting proteins from pollen grains of tomato (Saravanan and Rose, 2004). However, a protocol found suitable for a particular plant of a species may not be appropriate for the other. This is exemplified by studies that showed suitability of both Protocols 1 and 2 for different parts of *Arabidopsis thaliana* and banana leaves (Maldonado et al., 2008), while only Protocol 2 was found to be suitable for *Brassica* seeds (Carpentier et al., 2005; Devouge et al., 2007). Variations in spot number were also apparent in a study which used different methods of protein extraction (Saravanan and Rose, 2004).

It is evident from this study that it is almost improbable to define a single protein extraction protocol for different tissues that are endowed with developmental-specific metabolites. Similar views were proposed in earlier studies (Stalikas, 2007; Maldonado et al., 2008; Wu et al., 2014). Despite differential protein extraction efficacy from different tissues by Protocols 1, 2, and 3, majority of protein spots (using pH 3–10 IPG strips) remained concentrated in molecular weight/pI range from 36 to 175 kDa/4.5 to 7.5 (Figures 3A–F, Table 2) revealing dominance of acidic and high molecular weight range proteins for all the proteomes. Overall, this study demonstrated that Protocol 3 works well for seeds while, Protocol 1 for leaves and flowers of pigeonpea. Optimal standardization of tissue and species-specific extraction protocol for proteins for ensuring a development of a comprehensive proteome atlas is immensely needed for pigeonpea. It is anticipated that availability of a proteome atlas in a public domain would be a useful tool for identifying specific molecular traits that could potentially be exploited for developing abiotic-and/or biotic-stress resistant “smart” pigeonpea.

**Table 2:** **Number of protein spots with their corresponding gels in different pH ranges**.

	**Number of protein spots**								
	**pI 3.0–4.5**			**pI 4.5–7.0**			**pI 7.0–10.0**		
**Method**	**Seed**	**Leaves**	**Flowers**	**Seed**	**Leaves**	**Flowers**	**Seed**	**Leaves**	**Flowers**
Phenol	15	28	10	360	346	203	43	34	27
P+ TCA-Acetone	20	04	05	401	347	129	59	29	20
TCA-Acetone	17	05	00	102	88	42	12	18	10

## Conclusion

In the present study, three distinctive protein extraction protocols were employed for protein extraction, i.e., phenol extraction, TCA-acetone and P+ TCA-acetone protein extraction methods were evaluated in pigeonpea seeds, leaves and flowers by 2-DE electrophoresis. The results demonstrated the enhanced efficiency of the phenol based protocol as compared with TCA acetone extraction on the basis of protein yield, gel quality, spot numbers and quantities. This could be attributed to the fact that phenol is one of the strongest dissociating agents known to decrease molecular interactions between proteins and other materials, in addition to its selectivity as a solvent. The phenol extraction method gave the maximum protein yield and was particularly effective with recalcitrant tissues. Regarding the TCA-acetone extraction method, this precipitant is very effective, and allows instant elimination of proteolytic and other modifying enzymes. However, a disadvantage of TCA extracted proteins is that they are difficult to re-dissolve. In a comparative proteomic analysis, a real objective is to amplify the numbers of proteins that can be resolved, both Protocol 1 and 3 revealed themselves to be complementary since each protocol permits the extraction of specific proteins.

## Author Contributions

NS designed all experiments and contributed significantly to all wet and dry lab experiments; NJ and RK assisted in the wet lab experiments; NS, NJ, RK drafted the manuscript; AJ edited the manuscript; NKS and VR conceived the study, its design and coordination and helped to draft the manuscript. All authors read and approved the final manuscript.

### Conflict of Interest Statement

The authors declare that the research was conducted in the absence of any commercial or financial relationships that could be construed as a potential conflict of interest.
